# A Prospective Randomized Study of Brain Tissue Oxygen Pressure-Guided Management in Moderate and Severe Traumatic Brain Injury Patients

**DOI:** 10.1155/2015/529580

**Published:** 2015-08-27

**Authors:** Chien-Min Lin, Ming-Chin Lin, Sheng-Jean Huang, Cheng-Kuei Chang, Dan-Ping Chao, Tai-Ngar Lui, Hsin-I Ma, Ming-Ying Liu, Wen-Yuh Chung, Yang-Hsin Shih, Shin-Han Tsai, Hung-Yi Chiou, Mau-Roung Lin, Sen-Li Jen, Li Wei, Chung-Che Wu, En-Yuan Lin, Kuo-Hsing Liao, Yung-Hsiao Chiang, Wen-Ta Chiu, Jia-Wei Lin

**Affiliations:** ^1^Department of Neurosurgery, Taipei Medical University-Shuang Ho Hospital, Taipei, Taiwan; ^2^School of Medicine, Taipei Medical University, Taipei, Taiwan; ^3^Graduate Institute of Biomedical Informatics, College of Medical Science and Technology, Taipei Medical University, Taipei, Taiwan; ^4^Department of Surgery, National Taiwan University and Hospital, Taipei, Taiwan; ^5^Graduate Institute of Injury Prevention and Control, Taipei Medical University, Taipei, Taiwan; ^6^Department of Tourism and Leisure Management, China University of Technology, Taipei, Taiwan; ^7^Department of Neurosurgery, Taipei Medical University-Wan Fang Hospital, Taipei, Taiwan; ^8^Department of Neurological surgery, Tri-Service General Hospital and National Defense Medical Center, Taipei, Taiwan; ^9^Department of Neurosurgery, Taipei Veterans General Hospital, Taipei, Taiwan; ^10^School of Public Health, Taipei Medical University, Taipei, Taiwan; ^11^Department of Neurosurgery, Taipei Medical University Hospital, Taipei, > Taiwan; ^12^Department of Health, Executive Yuan, Taipei, > Taiwan

## Abstract

The purpose of this study was to compare the effect of PbtO_2_-guided therapy with traditional intracranial pressure- (ICP-) guided treatment on the management of cerebral variables, therapeutic interventions, survival rates, and neurological outcomes of moderate and severe traumatic brain injury (TBI) patients. From 2009 to 2010, TBI patients with a Glasgow coma scale <12 were recruited from 6 collaborative hospitals in northern Taiwan, excluding patients with severe systemic injuries, fixed and dilated pupils, and other major diseases. In total, 23 patients were treated with PbtO_2_-guided management (PbtO_2_ > 20 mmHg), and 27 patients were treated with ICP-guided therapy (ICP < 20 mmHg and CPP > 60 mmHg) in the neurosurgical intensive care unit (NICU); demographic characteristics were similar across groups. The survival rate in the PbtO_2_-guided group was also significantly increased at 3 and 6 months after injury. Moreover, there was a significant correlation between the PbtO_2_ signal and Glasgow outcome scale-extended in patients from 1 to 6 months after injury. This finding demonstrates that therapy directed by PbtO_2_ monitoring is valuable for the treatment of patients with moderate and severe TBI and that increasing PaO_2_ to 150 mmHg may be efficacious for preventing cerebral hypoxic events after brain trauma.

## 1. Introduction

Survival rates and outcomes for patients suffering from severe traumatic brain injury (TBI) depend on the severity of secondary cerebral insults [1, 2]. Previous studies have shown that cerebral ischemia, which may be caused by systemic hypotension, intracranial hypertension, impaired autoregulation, or hyperventilation, is a common and independent factor associated with neurological deterioration after injury [3–7]. Although no randomized trial has demonstrated an improved outcome for severe TBI patients provided with intracranial pressure (ICP) signal-guided treatment, current guidelines for severe TBI management recommend ICP monitoring to calculate and maintain cerebral perfusion pressure (CPP) and prevent cerebral ischemia and infarction [8]. However, certain studies have confirmed that CPP may not be correlated with cerebral blood flow and oxygen consumption in TBI patients [7, 9–12]. Cerebral ischemia and infarction may not be observed in patients with marginally low CPP, but these events may still occur, even with the maintenance of normal or supranormal CPP. In addition, certain CPP-guided treatments, such as the administration of vasopressor agents or fluid expanders, have been demonstrated to cause adverse effects, including respiratory distress syndrome, and were shown to offset the original benefits of these treatments on the outcome of severe TBI patients [13, 14].

A continuous supply of oxygen and glucose to brain tissue is important for maintaining a normal aerobic metabolism inside the brain cells. However, an excessive oxygen requirement and insufficient cerebral blood flow after trauma may cause cerebral hypoxia and transform a normal metabolism into an anaerobic condition [12, 15, 16]. The regional partial pressure of brain tissue oxygen (PbtO_2_) has been described as an independent, sensitive, and direct predictor of cerebral ischemia and hypoxia [9, 17, 18]. It has been reported that the incidence, frequency, and duration of cerebral hypoxic events, indicated by PbtO_2_ < 5, 10, or 15 mmHg, are significantly correlated with an unfavorable outcome in patients after trauma [7, 19–23]. The PbtO_2_ can be manipulated by adjusting the ICP and CPP or the fraction of inspired oxygen (FiO_2_). Decreasing ICP below 20 mmHg and increasing CPP above 60 mmHg, when guided by PbtO_2_ monitoring, were reported to relieve cerebral hypoxia simultaneously through intact cerebral autoregulation [19, 24]. However, the cardiopulmonary complications arising from ICP and CPP treatments should be managed carefully. Several studies have shown that elevating FiO_2_ can increase PaO_2_, reduce hypoxia, and reinstate the aerobic metabolism inside the brain cells with fewer cardiopulmonary complications [16, 25, 26].

The effects of TBI treatment using additional PbtO_2_ monitoring on cerebral hypoxia and patient outcome remain controversial, and evidence from randomized clinical studies is scant. Several investigators have reported that PbtO_2_-guided therapy significantly reduced cerebral hypoxic events and mortality rates and improved outcomes in severe TBI patients compared with historical controls [24, 27–29]. However, Martini and colleagues determined that severe TBI management guided by PbtO_2_ monitoring was associated with a poor neurological outcome and was an inefficient use of hospital resources [30]. Therefore, the purpose of this prospective randomized trial was to compare the effect of PbtO_2_-guided therapy (maintaining PbtO_2_ > 20 mmHg) with traditional ICP-guided treatment (maintaining ICP < 20 mmHg and CPP > 60 mmHg) on the management of cerebral variables, therapeutic interventions, survival rates, and neurological outcomes in moderate and severe TBI patients. The outcomes of TBI patients in this study were evaluated on both the GOS and the Glasgow Outcome Scale-Extended (GOSE).

## 2. Subjects and Methods

### 2.1. Subjects

This human study was approved by the Institutional Review Board (IRB) of 6 collaborative hospitals in northern Taiwan, including Taipei Veterans General Hospital, Tri-Service General Hospital, En Chu Kong Hospital, Taipei Medical University Hospital, Taipei Medical University-Wan Fang Hospital, and Taipei Medical University-Shuang Ho Hospital. The study was explained to patients by investigators, coinvestigators, or the nursing and research staff, and a statement of informed consent was signed by each patient. The medical records used in this study were also evaluated as part of an observational TBI registry with IRB approval.

Moderate TBI patients with Glasgow coma scale (GCS) scores of 9–12 and severe TBI patients with GCS scores <8 were considered for recruitment from January 2009 to December 2010. Eligible patients were aged 17–70 years. Patients were excluded if they presented with severe systemic injuries with hypotension or multiple trauma, a GCS = 3 with fixed and dilated pupils, or a supplied 100% FiO_2_ with saturation of arterial O_2_ below 93%. Patients whose families refused aggressive treatment, as well as those transferred from another institution more than 24 hours after injury, were also excluded, as were patients suffering from open gunshot or stab wounds, postcardiac pulmonary resuscitation or obvious hypoxia, or other major underlying diseases such as uremia, liver cirrhosis, congestive heart failure with pulmonary edema, or coagulopathy.

### 2.2. Patient Monitoring

Enrolled patients were randomized into ICP- and PbtO_2_-guided groups. Patients in the ICP group received an ICP monitor for use only in traditional ICP and CPP therapy. In the PbtO_2_-guided group, patients were treated using both ICP and PbtO_2_ monitors for combined FiO_2_ modulation with ICP and CPP management. An intraparenchymal or subdural ICP monitor (Codman electrode MicroSensor, Johnson and Johnson Medical, Ltd., USA) was used for monitoring the ICP signal in both groups. The ICP monitor was connected to an HP monitor (model 66s-M116A) through a pressure transducer and module (Codman neuromonitor interface control unit, 82-6605). In the PbtO_2_-guided group, an intraparenchymal brain tissue oxygen probe (LICOX REFIT2, Integra NeuroSciences, Ltd., England) was used for monitoring the PbtO_2_ signal. The PbtO_2_ signal was transmitted to an HP monitor through a transducer box and monitor cable (LICOX REF POM.BOX and LICOX REF NL950-MC-01, Integra NeuroSciences, Ltd., England). Both ICP and PbtO_2_ monitors were placed in the margin (2-3 cm) of necrotic brain tissue (hematoma) of TBI patients, located by the penumbra in preoperative brain computed tomography scans.

### 2.3. Patient Management

All patients were monitored by nursing staff in the NICU and were positioned in bed with a 30° head-up position. General monitoring included continual or intermittent assessment of mean arterial pressure, central venous pressure, electrocardiogram, pulse oximetry values, end-tidal CO_2_, and body temperature. Routine physical and pharmacological methods were adopted to prevent patient body temperature elevations above 37.5°C. Mechanical ventilation was adjusted to keep end-tidal CO_2_ between 30 and 35 mmHg. Osmotic modulation, sedation, and decompressive craniectomy were used to control intracranial hypertension.

The major differences between the ICP- and PbtO_2_-guided groups were the treatment goals. In the ICP-guided group, ICP was predominantly maintained at <20 mmHg, and CPP was maintained at >60 mmHg. However, in the PbtO_2_-guided group, PbtO_2_ was maintained at >20 mmHg, accompanied by ICP monitoring. Patients in the PbtO_2_-guided group were treated in compliance with standard procedures under 3 sets of conditions. If patients had intracranial hypertension (ICP > 20 mmHg) but a PbtO_2_ > 20 mmHg, then the primary strategy was the treatment of intracranial hypertension with mannitol, glycerol, colloid, sedatives, or decompressive craniectomy, which is similar to traditional ICP-guided management. Vasopressor agents and hyperventilation could also be used to raise CPP appropriately, while controlling PbtO_2_.

If patients had marginal cerebral hypoxia (PbtO_2_ < 20 mmHg) but ICP at <20 mmHg, PbtO_2_ was normalized with a 100% FiO_2_ challenge for 5 minutes. If PbtO_2_ was increased by the FiO_2_ challenge, then FiO_2_ was slowly tapered while maintaining PbtO_2_. The 100% FiO_2_ challenge was limited to 5 hours or less to avoid oxygen intoxication. If 100% FiO_2_ was needed for longer than 5 hours, CPP was elevated up to 80 mmHg, and arterial carbon dioxide pressure (PaCO_2_) was elevated to 40 mmHg to replace long-term high-percentage FiO_2_ administration. If PbtO_2_ was not increased by the FiO_2_ challenge, then CPP and PaCO_2_ could also be raised to resolve continuous cerebral ischemia after confirmation by brain computed tomography that the sensor tip was in place, and there was no evidence of heart failure or lung problems (pulmonary edema or acute respiratory distress syndrome).

Finally, if both intracranial hypertensive (ICP > 20 mmHg) and marginally cerebral hypoxic (PbtO_2_ < 20 mmHg) events occurred simultaneously, then normalization of PbtO_2_ was the most important strategy.

Each of the 6 collaborative hospitals offered training courses in the standard protocol for multisite principal investigators, coinvestigators, and research assistants to maintain optimal patient management and avoid cluster effects caused by differences in equipment, faculty, and patient sources.

### 2.4. Data Collection

Collected patient data included age, the initial GCS score, recruiting year, body mass index, injury etiology, the pathological status diagnosed by brain computed tomography, and operative status. Cerebral variables, such as GCS score, ICP, CPP, PbtO_2_, PaO_2_, and PaCO_2_, were noted during the first 5 days in the NICU, as well as the therapeutic administration of drugs such as mannitol, glycerol, colloids, vasopressor agents, and sedatives. Intracranial hypertension was indicated as ICP > 20 mmHg, whereas cerebral ischemia was defined as CPP < 60 mmHg. The CPP was calculated as the mean arterial pressure minus the ICP. Cerebral hypoxia was defined as PbtO_2_ < 15 mmHg and was measured only in the PbtO_2_ group. In addition, the survival rates and outcomes at 1, 3, and 6 months following injury were evaluated using the GOS and GOSE questionnaires. The collaborative hospitals held regular meetings to verify that all eligible patients were enrolled and that the data recording was complete.

### 2.5. Statistical Analysis

After the data were cleaned and checked for completeness, we used an *χ* ^2^ test and an independent Student's *t* test to compare categorical and continuous data between the ICP- and PbtO_2_-guided groups. Categorical variables included age, initial GCS score, recruiting year, etiology, pathological diagnosis, and operative status, as well as the appearance of intracranial hypertension, cerebral ischemia, therapeutic interventions, survival, and favorable outcome. Continuous variables included the mean age, initial GCS score, and cerebral monitoring over 5 days in the NICU; the data were expressed as means ± SD. Linear regression was used to further evaluate the relationship between PbtO_2_ and GOS and GOSE scores. All statistical calculations were performed using SPSS, version 17.0 (SPSS, Chicago, IL, USA). Differences were considered statistically significant at *P* < 0.05.

## 3. Results

Twenty-seven TBI patients were treated with traditional ICP-guided therapy (mean age 53.3 ± 20.1 y), whereas 23 patients were treated with PbtO_2_-guided management (mean age 53.7 ± 19.4 y).  Table 1 shows the demographic data for both ICP- and PbtO_2_-guided groups. The distribution of age, initial GCS score, recruiting year, body mass index, etiology, pathological diagnosis, and operative status was similar across the 2 groups.

**Figure 1:**
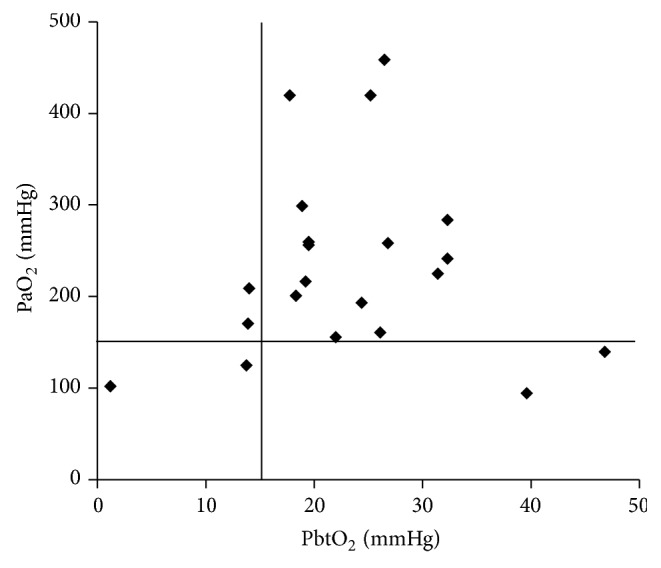
Distribution of average PaO_2_ and PbtO_2_ signals of TBI patients guided by PbtO_2_ monitoring within 5 ICU days.

Cerebral monitoring variables over 5 days in the NICU are shown in Table 2. In the ICP-guided group, mean ICP was significantly higher (*P* = 0.017), and the intracranial hypertensive events (ICP > 20 mmHg) were almost 5 times more frequent (22.2% versus 4.3%) than in the PbtO_2_-guided group. The average CPP was significantly higher (*P* = 0.013) in PbtO_2_-guided patients compared with ICP-guided patients, although these signals were >60 mmHg in both groups. The mean GCS score and PaCO_2_ showed no difference between the ICP- and PbtO_2_-guided groups. The mean PaO_2_ was significantly elevated in the PbtO_2_-guided group compared with the ICP-guided group (*P* = 0.033).  Figure 1 shows that the mean PaO_2_ should be adjusted to >150 mmHg to prevent cerebral hypoxic events (PbtO_2_ < 15 mmHg) in patients with moderate to severe TBI. Therapeutic interventions were comparable across both treatment groups, as shown in Table 3.

**Figure 2:**
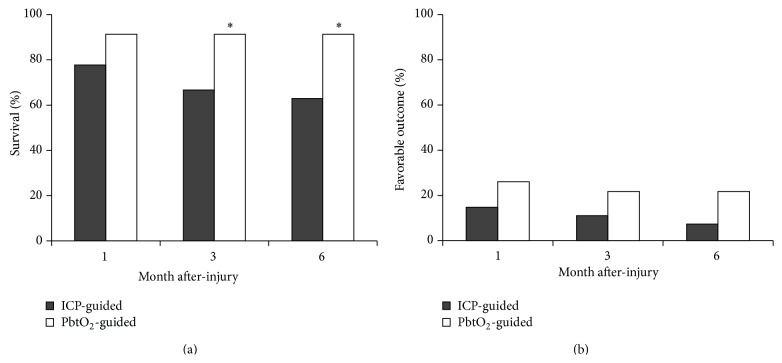
Ratio of survival (a) and favorable outcome (b) of TBI patients in ICP and PbtO_2_ guided groups at one, three, and six months after injury. ^*∗*^ *P* < 0.05 (ICP guided group versus PbtO_2_ guided group at the same month).

In Table 4, we provided the mortality rate of each group. Survival rates were significantly higher in patients guided by PbtO_2_ monitoring at 3 and 6 months postinjury compared with those guided by ICP monitoring (Figure 2(a)). However, a favorable outcome was observed in <30% of patients in either group at any point after injury (Figure 2(b)). Mean GOS scores were 2.2–2.3 and 2.6–2.7, and mean GOSE scores were 2.4–2.6 and 3.0–3.2 in the ICP- and PbtO_2_-guided groups, respectively, from 1 to 6 months postinjury. Although no differences were significant in favorable outcome rates across the 2 groups, patients in the PbtO_2_-guided group had a 1.8–2.9 times more favorable outcome from 1 to 6 months postinjury than patients in the ICP-guided group.

**Figure 3:**
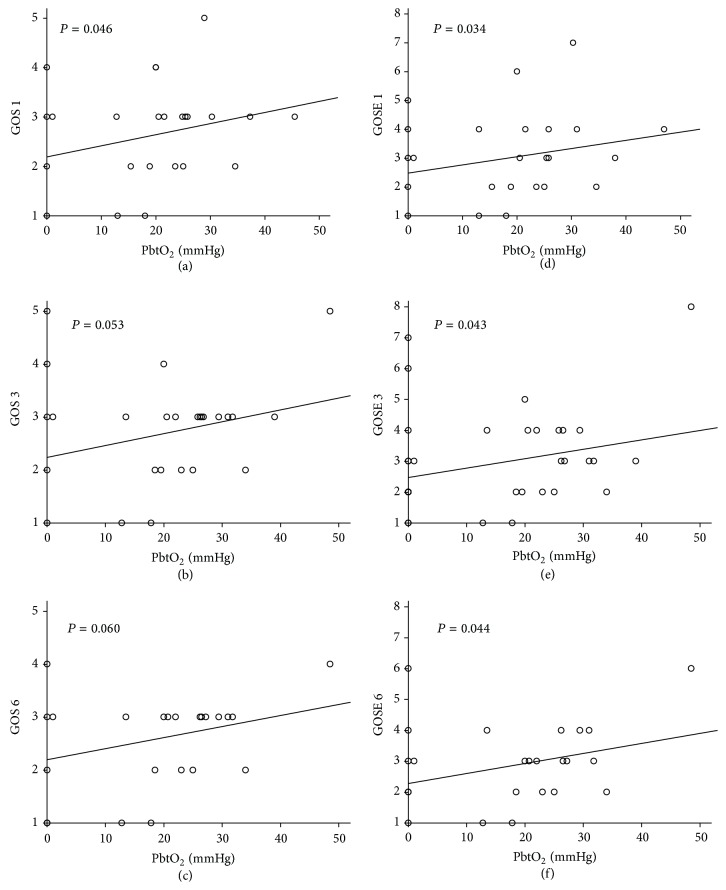
Correlation of PbtO_2_ signal with GOS ((a), (b), (c)) and GOSE ((d), (e), (f)) scores of TBI patients at one ((a), (d)), three ((b), (e)), and six ((c), (f)) months after injury.

Figure 3 shows the correlation between the PbtO_2_ signal and the outcome scale of TBI patients at 1, 3, and 6 months after injury. The PbtO_2_ signals were significantly correlated with GOS scores at 1 and 3 months postinjury (Figures 3(a) and 3(b)) and with GOSE scores from 1 to 6 months postinjury (Figures 3(d)–3(f)). However, at 6 months postinjury, the correlation between PbtO_2_ signals and GOS scores (Figure 3(c)) was still marginally significant (*P* = 0.060).

## 4. Discussion

Several studies have suggested that PbtO_2_ is an independent factor related to the neurological outcome of severe TBI patients and weakly correlated with ICP or CPP signals [7, 31]. Other investigators have reported a positive correlation between PbtO_2_ and CPP under a specific range of CPP [19, 32]. Although our results show that both ICP and CPP were managed in the reference range in most patients, significantly lower ICP and higher CPP were observed in patients treated with PbtO_2_ monitoring compared with those treated with ICP monitoring alone. Treatment interventions were comparable between the 2 groups. Intracranial hypertensive events (ICP > 20 mmHg) in the PbtO_2_-guided group were rare in our study, which was similar to the previous results by Meixensberger et al. [24].

Previous studies have demonstrated that a cerebral hematoma that impairs blood flow and oxygen delivery into the brain tissue and induces secondary ischemia can progress with time after an intracerebral hemorrhage [33–35]. Furthermore, ischemia is a cause of brain edema surrounding the hematoma region [34, 35]. Irrespective of whether maintaining cerebral oxygen consumption could reduce ischemia-induced edema, further improving CPP and normalizing ICP, even when these signals have already been controlled, is an important issue that requires further investigation.

Fewer patients (*n* = 1; 4.3%) in the current study had pulmonary complications in the PbtO_2_-guided group compared with the ICP-guided group (*n* = 3; 11.1%). Stiefel et al. suggested that treating severe TBI patients with cause-specific management might result in improved survival rates and improved outcomes compared with treating ICP or CPP alone [27]. Increasing FiO_2_ in patients with severe TBI has been studied as a strategy for achieving elevated PaO_2_ and adequate PbtO_2_ to avoid cerebral hypoxia and further improve outcomes by returning the cerebral metabolism to aerobic conditions [16, 25, 26, 32, 36–38]. As shown in this study, the mean PaO_2_ increased in PbtO_2_-guided patients. Therefore, a combination of FiO_2_ modulation with traditional ICP and CPP treatment may simultaneously prevent hypoxia and other secondary complications, especially in pulmonary events. We also suggest that the PaO_2_ targets in moderate and severe TBI patients should be different from those in general neurological patients. In moderate and severe TBI patients, PaO_2_ must be adjusted to a value >150 mmHg, in contrast with the PaO_2_ standard (PaO_2_ > 60 mmHg), to prevent cerebral hypoxic events after trauma.

Our results indicate that patients guided by PbtO_2_ monitoring had increased survival rates at 3 and 6 months postinjury. Nevertheless, mean GOS scores were <3, and favorable outcome rates were <30% in both groups at all the time points postinjury. A trend emerged toward greater favorable outcomes (1.8–2.9 times greater) in PbtO_2_-guided patients versus ICP-guided patients from 1 to 6 months after injury. These results are similar to those of other studies that observed a positive trend toward more favorable outcomes in severe TBI patients treated with PbtO_2_-guided monitoring [24, 27]. The small difference in favorable outcomes between groups in our study was possibly due to most patients (>70%) having a TBI that was too severe to permit recovery. Spiotta et al. reported a study with more patients (*n* = 123) and noted a significantly improved short-term outcome in PbtO_2_-guided patients [29]. Therefore, the small sample may be another reason that differences in favorable outcome were undetectable for patients in our study. Despite the differences in favorable outcomes between the 2 groups in the study, positive correlations were noted between PbtO_2_ signals and outcome scales, especially in GOSE scores, at 1, 3, and 6 months postinjury.

This study has several strengths and limitations. This was the first prospective randomized clinical trial studying the application of PbtO_2_ monitoring for patients suffering from moderate to severe TBI. Additional strengths were shown in the collaboration among the 6 hospitals in northern Taiwan, which managed unselected brain-trauma patients, and held regular meetings to standardize the collection and recording of data. One limitation was the small number of patients enrolled, which may have resulted in an inability to detect differences in patient outcomes between the ICP- and PbtO_2_-guided groups. However, the positive correlation between PbtO_2_ signal and GOSE score reported here reflects the importance of PbtO_2_ monitoring in TBI patients. Another limitation was that PbtO_2_ and FiO_2_ signals could not be compared across groups. The PbtO_2_ was not monitored in the ICP-guided group, and FiO_2_ was adjusted only according to PbtO_2_ signals in the PbtO_2_-guided group during the first 5 days in the NICU, rather than continually recorded. Hence, cerebral oxygen consumption in the PbtO_2_-monitored patients was increased in accordance with increased PaO_2_ in this study and in previously published studies [26, 32].

In conclusion, this study demonstrates that (1) there was an increase in PbtO_2_ related to increase of PaO_2_. (2) The PbtO_2_ signals demonstrate a close correlation with patient outcomes from 1 to 6 months postinjury. Other than increase of PaO_2_, hemoglobin transfusion, decreasing oxygen demand (increased sedation, paralysis, and barbiturates use), and increased CO_2_ (if ICP is controlled) can improve cerebral hypoxia. Therefore, we propose that PbtO_2_ control is correlated with increase of PaO_2_ and that therapy directed by PbtO_2_ monitoring may be valuable in treating patients with moderate or severe TBI. In addition, increasing PaO_2_ above 150 mmHg seems to efficaciously prevent cerebral hypoxic events after trauma. The mechanism and effects of PbtO_2_ manipulation on well-controlled ICP (<20 mmHg) and CPP (>60 mmHg) TBI patients require further investigation.

Due to the small sample size in this study, we concluded that PbtO_2_-monitoring therapy might be beneficial in clinical care for managing moderate to severe brain injured patients. Firm conclusion shall be drawn in a larger and adequately designed study in the future.

## Figures and Tables

**Table 1:** Demographic characteristics of TBI patients guided by ICP and PbtO_2_ monitoring.

	ICP-guided	PbtO_2_-guided	*P*
Number	27	23	
Age			
<40 y/o	8 (29.6)	6 (26.1)	0.781
≧40 y/o	19 (70.4)	17 (73.9)	
Average age	53.3 ± 20.1	53.7 ± 19.4	0.938
Initial GCS			
3–8	19 (70.4)	17 (73.9)	0.781
9–12	8 (29.6)	6 (26.1)	
Average GCS	6.9 ± 2.6	7.1 ± 2.7	0.791
Recruiting year			
2009	12 (44.4)	10 (43.5)	0.945
2010	15 (55.6)	13 (56.5)	
BMI	22.7 ± 4.3	23.9 ± 4.7	0.441
Etiology			
Traffic accident	15 (55.6)	13 (56.5)	0.945
Fall	12 (44.4)	10 (43.5)	
Pathological diagnosis			
SDH	20 (74.1)	14 (60.9)	0.318
EDH	2 (7.4)	5 (21.7)	0.145
SAH	11 (40.7)	10 (43.5)	0.845
ICH	11 (40.7)	9 (39.1)	0.908
IVH	2 (7.4)	1 (4.3)	0.650
Contusion	3 (11.1)	4 (17.4)	0.524
Skull fracture	3 (11.1)	5 (21.7)	0.307
Uncal herniation	1 (3.7)	0 (0.0)	0.351
Brain swelling	1 (3.7)	0 (0.0)	0.351
Operation			
Craniotomy	25 (92.6)	21 (91.3)	0.867
Craniectomy	22 (88.0)	18 (90.0)	0.832

**Table 2:** Cerebral variables of TBI patients guided by ICP and PbtO_2_ monitoring in the ICU.

	ICP-guided	PbtO_2_-guided	*P*
GCS	6.2 ± 2.3	6.7 ± 2.4	0.424
ICP (mmHg)	17.9 ± 13.0	10.1 ± 4.7	0.017^*∗*^
ICP > 20 mmHg	6 (22.2)	1 (4.3)	0.069
CPP (mmHg)	73.5 ± 10.6	83.1 ± 11.5	0.013^*∗*^
CPP < 60 mmHg	1 (3.7)	0 (0.0)	0.351
PbtO_2_ (mmHg)	—	22.8 ± 9.9	—
PbtO_2_ < 15 mmHg	—	5 (21.7)	—
PaO_2_ (mmHg)	174.3 ± 73.9	232.4 ± 98.7	0.033^*∗*^
PaCO_2_ (mmHg)	33.3 ± 5.0	33.1 ± 5.7	0.867

^*∗*^ *P* < 0.05.

**Table 3:** Therapeutic interventions of TBI patients guided by ICP and PbtO_2_ monitoring.

	ICP-guided	PbtO_2_-guided	*P*
Mannitol	19 (70.4)	15 (65.2)	0.697
Glycerol	7 (25.9)	8 (34.8)	0.496
Colloid	17 (63.0)	14 (60.9)	0.879
Vasopressors	7 (25.9)	2 (8.7)	0.114
Sedatives	16 (59.3)	18 (78.3)	0.151

**Table 4:** The mortality rate of each group.

Initial GCS	ICP-guided	PbtO_2_-guided
3–8	9	1
9–12	1	0
Total	**10**	**1**
